# Analysis of factors influencing vascular calcification in peritoneal dialysis patients and their impact on long-term prognosis

**DOI:** 10.1186/s12882-024-03582-2

**Published:** 2024-05-07

**Authors:** Qianying Cao, Yanan Shi, Xiaohui Liu, Fan Yang, Xiangnan Li, Zhongxin Li

**Affiliations:** 1 Department of Nephrology, Beijing Luhe Hospital, Capital Medical University, No.82 Xinhua South Road, Tongzhou District, Beijing, 101149 China; 2 Department of Nephrology, Space Center Hospital, Beijing, China

**Keywords:** Peritoneal dialysis, Vascular calcification, Sclerostin, All-cause mortality, Risk factors

## Abstract

**Background:**

This study aims to investigate the influencing factors of vascular calcification in peritoneal dialysis (PD) patients and its relationship with long-term prognosis.

**Methods:**

This retrospective cohort study included chronic kidney disease patients undergoing peritoneal dialysis at the Peritoneal Dialysis Center of Beijing Luhu Hospital, Capital Medical University, from January 2019 to March 2019. Demographic and clinical laboratory data, including serum sclerostin (SOST), calcium (Ca), phosphate (P), serum albumin (ALB), and intact parathyroid hormone (iPTH) levels, were collected. Abdominal aortic calcification (AAC) was assessed using abdominal lateral X-ray examination to determine the occurrence of vascular calcification, and patients were divided into the AAC group and Non-AAC group based on the results.

**Results:**

A total of 91 patients were included in the study. The AAC group consisted of 46 patients, while the Non-AAC group consisted of 45 patients. The AAC group had significantly older patients compared to the non-AAC group (*P* < 0.001) and longer dialysis time (*P* = 0.004). Multivariable logistic regression analysis indicated that risk factors for vascular calcification in PD patients included dialysis time, diabetes, hypertension, and SOST. Kaplan–Meier survival analysis showed that the AAC group had a significantly higher mortality rate than the non-AAC group (χ2 = 35.993, *P* < 0.001). Multivariable Cox regression analysis revealed that dialysis time, diabetes and AAC were risk factors for all-cause mortality in peritoneal dialysis patients.

**Conclusion:**

Longer dialysis time, comorbid diabetes, comorbid hypertension, and SOST are risk factors for vascular calcification in PD patients. Additionally, AAC, longer dialysis time, and comorbid diabetes are associated with increased risk of all-cause mortality in peritoneal dialysis patients.

## Introduction

Chronic kidney disease (CKD) has become a global public health issue. CKD-mineral and bone disorder (CKD-MBD) is prevalent in patients with moderate to advanced CKD and is closely associated with pathological fractures, vascular calcification, acute cardiovascular events, and mortality rates [1]. Peritoneal dialysis (PD) is a common renal replacement therapy for patients with late-stage CKD. However, over time, PD patients commonly experience vascular calcification, which significantly impacts their long-term prognosis. Studies have reported that the prevalence of coronary artery and aortic calcification in CKD stages 3–5 patients ranges from 40 to 60%, and in CKD stage 5 patients, it can be as high as 80% to 90% [2].

There are several methods for assessing vascular calcification in PD patients, with computed tomography (CT) being the gold standard for evaluating vascular calcification. However, X-ray radiography is a cost-effective and lower radiation exposure alternative method [3]. Bellasi et al. [4] found a good correlation between abdominal aortic calcification (AAC) detected by lateral abdominal X-ray and cardiovascular calcification scores, with a sensitivity of 67% and specificity of 91%. Moreover, assessing abdominal aortic calcification can also help determine the extent of coronary artery calcification [5]. The Kidney Disease Improving Global Outcomes (KDIGO) guidelines recommend using lateral abdominal X-ray in CKD stages 3–5 patients as an alternative method to CT imaging for detecting vascular calcification [6].

Vascular calcification has a significant impact on the long-term prognosis of PD patients. Studies have shown that vascular calcification is closely associated with increased cardiovascular event rates, progression of vascular disease, and higher risk of mortality [7]. A large prospective study conducted in 47 dialysis centers in Europe, including 1,084 hemodialysis (HD) patients, found that AAC was an independent predictor of all-cause mortality and non-fatal cardiovascular events in dialysis patients [8]. Previous studies in the PD population have identified AAC as a risk factor for coronary artery calcification and cardiovascular disease [9]. Therefore, investigating the risk factors for vascular calcification in PD patients is of significant clinical value for understanding patient conditions.

Vascular calcification is a complex pathological process involving intimal and medial arterial calcification. It is associated with various factors, including metabolic disorders, inflammatory responses, vascular injury, and abnormalities in phosphate metabolism. PD patients often experience disturbances in phosphate metabolism, such as hyperphosphatemia and hypocalcemia, due to impaired renal function, which may be a major trigger for vascular calcification. In addition to disturbances in phosphate metabolism, recent studies have found that factors such as serum sclerostin (SOST), calcium, phosphate, and intact parathyroid hormone (iPTH) levels may be closely associated with the occurrence and progression of vascular calcification in PD patients. SOST, a protein produced by bone cells, inhibits the process of bone formation and may play a significant role in vascular calcification. Elevated levels of sclerostin are associated with increased risk of vascular calcification. Comprehensive analysis and evaluation of the influencing factors on vascular calcification in PD patients, as well as their impact on long-term prognosis, are of great significance in improving the survival quality and prognosis of PD patients. By studying the pathogenesis and related influencing factors of vascular calcification in depth, we can provide more effective personalized treatment strategies for PD patients, reduce the occurrence of complications, and improve patient survival rates and quality of life. The aim of this study is to investigate the correlation between serum sclerostin levels and other serological markers with the occurrence of abdominal aortic calcification in maintenance PD patients and analyze the relationship between these influencing factors and the survival rate of PD patients.

## Materials and methods

### Patients and study design

**Fig. 1:**
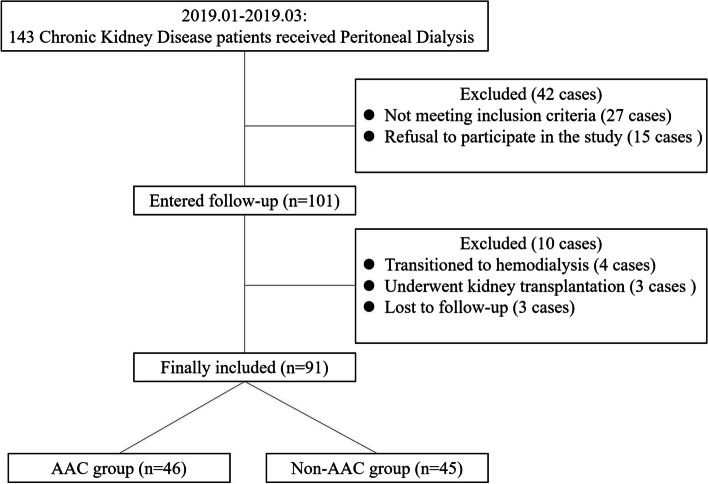
Flowchart of patient enrollment

This retrospective cohort study selected chronic kidney disease patients who underwentcontinuous ambulatory peritoneal dialysis at the Peritoneal Dialysis Center of Beijing Luhe Hospital, Capital Medical University, from January 2019 to March 2019 as the study subjects. The shortest and longest duration of peritoneal dialysis at the enrollment were 12 months and 138 months, respectively. Inclusion criteria were as follows: ① age ≥ 18 years; ② dialysis duration > 6 months; ③ regular follow-up with complete medical records. Exclusion criteria were as follows: ①autoimmune diseases and/or corticosteroid therapy during follow-up; ② concurrent diseases affecting bone metabolism, including multiple myeloma, etc.; ③ primary parathyroid disease or history of parathyroidectomy. After screening, a total of 91 patients were included in the study (Fig. 1). The patients all used peritoneal dialysis solution produced by Baxter (China) Investment Co., Ltd. (Ca2 + 1.25 mmol/L and/or 1.75 mmol/L). Dialysis methods included continuous ambulatory peritoneal dialysis and daytime ambulatory peritoneal dialysis, with fluid exchange performed 3–5 times per day, 1500–2000 mL each time, and a dialysis dose of 6–10 L per day. Follow-up was conducted until May 2023, with a total duration of 48 months, and the primary endpoint was all-cause mortality. The time and causes of death were recorded based on patient death cases. Patients who discontinued peritoneal dialysis during the follow-up period were also recorded for their withdrawal time and reasons, while maintaining follow-up.

This study was approved by the Ethics Committee of Beijing Luhe Hospital, Capital Medical University (Ethics Approval Number: 2019-LHKY-018–01).

### Clinical data collection

Clinical data including age, gender, height, weight, duration of dialysis, and cardiovascular disease history were collected from the patients. Information regarding comorbidities such as diabetes and hypertension was also recorded. The urea clearance rate (Kt/V) was calculated using the formula: Kt/V = -In(R—0.008*t + 4—3.5*R) * UF/W, where K represents the clearance rate, t denotes the treatment time, V represents the urea distribution volume, R represents the pre-dialysis urea nitrogen divided by the post-dialysis urea nitrogen, UF represents the ultrafiltration rate (Is), and W represents the dry body weight in kilograms.

### Serum samples

Venous blood samples were collected from all PD patients in a fasting state before dialysis. Calcium (Ca), phosphate (P), and serum albumin (ALB) levels were measured using the fully automated biochemical analyzer Hitach 7600–210 from Switzerland. The intact parathyroid hormone (iPTH) was measured using a Roche electrochemiluminescence immunoassay analyzer (Roche Diagnostics,). Sclerostin, a marker for bone sclerosis, was detected using an enzyme-linked immunosorbent assay (ELISA) kit obtained from a US-based company (American R&D Company).

### Vascular calcification assessment

Assessment of vascular calcification was performed based on abdominal aortic calcifications (AAC). Abdominal imaging was conducted using the Revolution XR/D DR X-ray machine, with patients positioned laterally. Fasting patients underwent lateral abdominal Z-line radiography, covering the region from T12-L1 to L4-L5 intervertebral spaces. To determine the AAC score, Kauppila’s approach was employed, which divided the aorta’s anterior and posterior walls into four sections adjacent to the lumbar vertebrae L1-L4 [10]. The four segments were divided into 8 parts according to the midline of each intervertebral disc and scored separately: Those who scored 0 were divided into the non-AACgroup; while those who scored ≥ 1 were assigned to the AAC group. The score were accessed by two experienced radiologists in a double-blind manner and averaged.

### Statistical analysis

Statistical analysis of the data was performed using SPSS 22.0 software. Normally distributed continuous variables were presented as mean ± standard deviation, and between-group comparisons were conducted using t-tests. Non-normally distributed continuous variables were presented as median (P25, P75), and between-group comparisons were performed using the Mann–Whitney U test. Categorical variables were presented as frequencies (%), and between-group comparisons were assessed using the chi-square test. Single-factor logistic regression analysis was employed to identify statistically significant indicators of differences between the two groups, and odds ratios (OR) were calculated. Variables with statistical significance were included in the multivariable logistic regression model. Kaplan–Meier survival curves were used to analyze the differences in patient survival between groups. A univariate Cox regression analysis was conducted to identify potential prognostic factors influencing survival outcomes. Variables that demonstrated statistical significance were subsequently included in the multivariable Cox regression analysis to determine the risk factors associated with overall mortality. A *p*-value of < 0.05 was considered statistically significant for all analyses.

## Results

### Baseline characteristics

A total of 91 PD patients were included, with a mean age of 59.62 ± 11.08 years. The incidence of AAC was 50.55%. Among them, there were 37 males (40.7%) with a median age of 59.62 (57.31, 61.92) years and a median dialysis duration of 46.00 (27.00, 67.00) months. There were 47 patients (51.6%) with concomitant diabetes and 41 patients (45.1%) with concomitant hypertension. The AAC group consisted of 46 patients, while the non-AAC group consisted of 45 patients. The AAC group had significantly higher age compared to the non-AAC group (64.11 ± 7.50 years vs. 55.02 ± 12.29 years, *P* < 0.001), longer dialysis duration [57.00 (35.50, 77.00) months vs. 40.00 (25.50, 56.50) months, *P* = 0.004], a higher proportion of patients with concomitant hypertension (33 cases vs. 8 cases, *P* < 0.001), and a higher proportion of patients with concomitant diabetes (28 cases vs. 9 cases, *P* < 0.001) (Table 1).

**Table 1:** Comparison of general characteristics between the AAC group and the control group (*N* = 91)

Items	Total (*n* = 91)	Non-ACC group (*n* = 45)	AAC group (*n* = 46)	T/Z/χ2	*P* value
Age (year)	59.62 ± 11.08	55.02 ± 12.29	64.11 ± 7.50	-4.245	< 0.001
Male (n,%)	37(41)	22(49)	15(33)	2.499	0.114
Dialysis time (month)	46.00(27.00,67.00)	40.00(25.50,56.50)	57.00(35.50,77.00)	-2.843	0.004
BMI (kg/m^2^)	23.56(22.64,25.26)	23.83(23.01,25.26)	23.44(22.18,25.58)	-1.139	0.255
Creatinine	837.45 ± 220.32	845.11 ± 244.61	829.96 ± 196.12	0.326	0.745
BUN	23.00(17.00,26.00)	23.00(17.00,26.00)	22.00(17.00,26.00)	-0.493	0.622
Uric acid	359.00(318.00,398.00)	354.00(319.50,394.00)	364.00(313.75,416.25)	-0.560	0.576
Calcium	2.17 ± 0.19	2.17 ± 0.20	2.17 ± 0.18	-0.048	0.962
Phosphorus	1.71 ± 0.48	1.69 ± 0.44	1.72 ± 0.51	-0.221	0.826
Albumin	42.80(41.20,44.60)	43.00(41.00,44.00)	43.00(41.00,45.00)	-0.750	0.453
iPTH	204.00(139.00,321.00)	199.00(131.50,332.50)	212.50(154.00,299.50)	-0.345	0.730
Hemoglobin	116.57 ± 10.69	117.76 ± 8.95	115.41 ± 12.14	1.049	0.299
Cholesterol	4.22 ± 0.83	4.23 ± 0.88	4.21 ± 0.79	0.091	0.928
ALP	76.00(62.00,92.00)	76.00(62.50,96.00)	74.00(62.00,91.25)	-0.687	0.492
LDL	2.43 ± 0.61	2.44 ± 0.67	2.42 ± 0.55	0.172	0.864
Ferritin	382.00(226.00,528.00)	426.00(205.50,548.00)	368.00(240.75,463.25)	-0.325	0.745
Comorbidity					
Diabetes (n,%)	41(45)	8(18)	33(70)	26.756	< 0.001
Hypertension (n,%)	37(41)	9(20)	28(61)	15.748	< 0.001
KT/V	1.98(1.89,2.10)	2.01(1.90,2.12)	1.94(1.77,2.10)	-1.454	0.146
SOST	276.38(196.93,402.52)	228.00(157.00,313.00)	343.50(250.50,470.00)	-3.826	< 0.001

*BMI* Body mass index, *BUN* blood urea nitrogen, *iPTH* intact parathyroid hormone, *ALP* alkaline phosphatase, *LDL* low-density lipoprotein, *KT/V* urea clearance index, *SOST *sclerostin,* AAC *abdominal aortic calcification

In all patients with concomitant diabetes, diabetes was the cause of chronic kidney disease. As glucose dialysate was used during peritoneal dialysis, these patients require additional glucose-lowering therapy.

### Analysis of factors influencing vascular calcification

Logistic regression analysis was performed to identify risk factors for vascular calcification in PD patients. Significant variables identified in the univariate regression analysis were further included in the multivariate regression analysis. The multivariate logistic regression analysis revealed that the risk factors for vascular calcification in PD patients included dialysis duration (OR = 1.025, 95% CI: 1.000–1.050, *P* = 0.047), diabetes (OR = 19.752, 95% CI: 4.490–86.897, *P* = 0.000), hypertension (OR = 12.947, 95% CI: 2.887–58.061, *P* = 0.001), and SOST levels (OR = 1.006, 95% CI: 1.000–1.011, *P* = 0.038) (Table 2).

**Table 2:** Logistic regression analysis of factors influencing abdominal aortic calcification in hemodialysis patients

Items	Univariate logistic regression analysis				Multivariable logistic regression analysis			
	B	OR	95%CI	*P*	B	OR	95%CI	*P*
Gender (Male)	-0.681	0.506	0.216–1.183	0.116	-	-	-	-
Age	0.095	1.099	1.043–1.158	0.000*	0.077	1.080	0.986–1.182	0.097
Dialysis time	0.027	1.028	1.009–1.047	0.004*	0.025	1.025	1.000–1.050	0.047*
Diabetes	-2.463	0.085	0.031-.231	0.000*	2.983	19.752	4.490–86.897	0.000*
Hypertension	-1.828	0.161	0.063-.412	0.000*	2.561	12.947	2.887–58.061	0.001*
Phosphorus	0.098	1.103	0.467–2.605	0.823	-	-	-	-
Calcium	0.053	1.055	0.124–9.005	0.961	-	-	-	-
Albumin	0.031	1.031	0.892–1.192	0.676	-	-	-	-
Triglycerides	-0.368	0.692	0.441–1.085	0.109	-	-	-	-
Cholesterol	-0.319	0.727	0.438–1.207	0.218	-	-	-	-
ALP	-0.003	0.997	0.985–1.010	0.656	-	-	-	-
LDL	-0.572	0.564	0.280–1.137	0.110	-	-	-	-
Hemoglobin	-0.021	0.979	0.941–1.019	0.297	-	-	-	-
Ferritin	0.000	1.000	0.998–1.001	0.674	-	-	-	-
iPTH	0.000	1.000	0.998–1.002	0.981	-	-	-	-
Kt/V	-2.161	0.115	0.013–1.032	0.053	-	-	-	-
SOST	0.007	1.007	1.003–1.011	0.001*	0.006	1.006	1.000–1.011	0.038*

*ALP* Alkaline phosphatase, *LDL* Low-density lipoprotein, *iPTH* intact parathyroid hormone, *Kt/V* urea clearance index, *SOST* sclerostin

^*^with significant differences

### Survival analysis

**Fig. 2:**
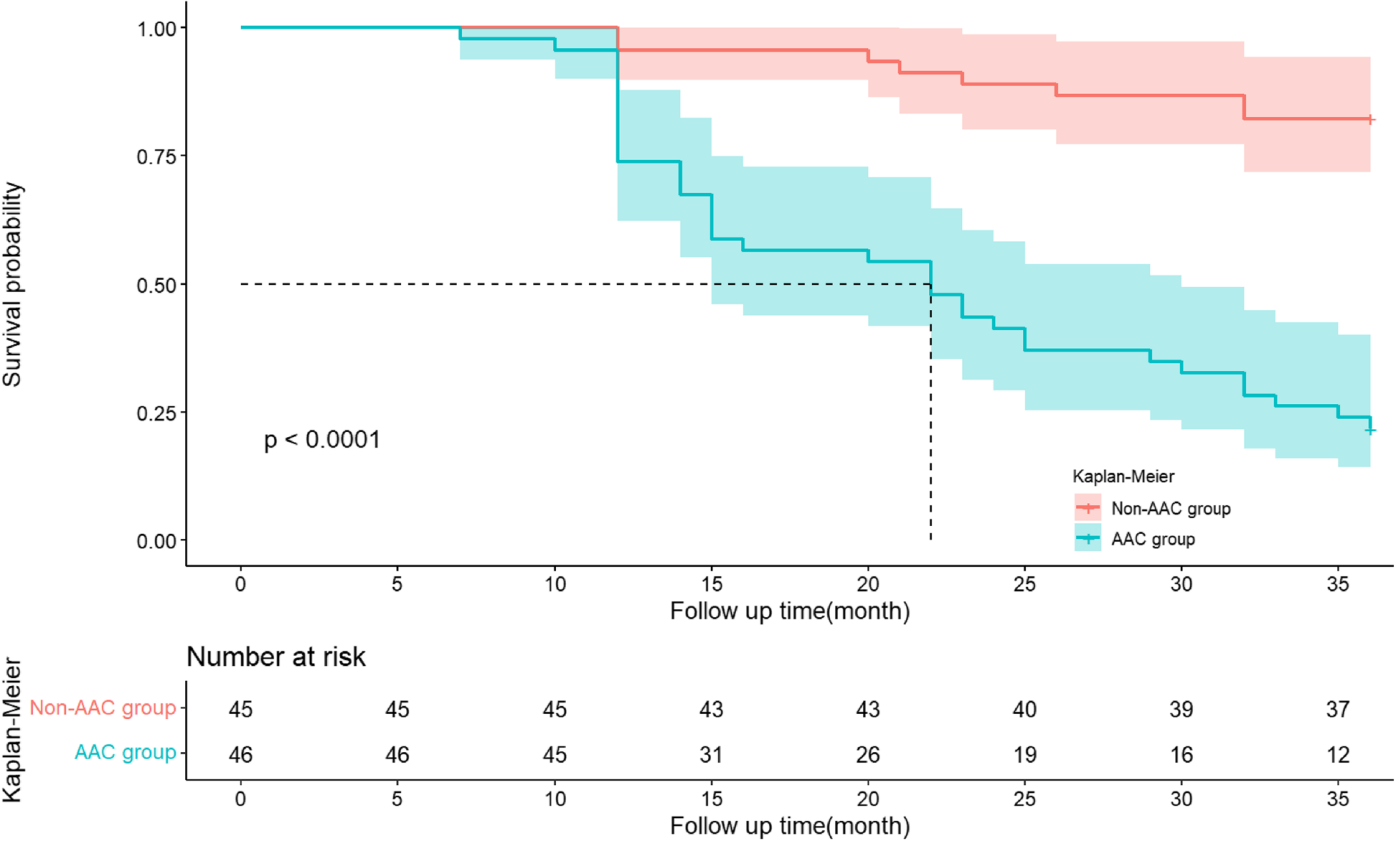
The differences in the survival time distribution of peritoneal dialysis patients were tested using the log-rank test. There was a statistically significant difference in the overall survival time distribution between the AAC group and the Non-AAC group, with a chi-square value of 35.993 and a *P*-value of < 0.001

A total of 91 patients had no loss to follow-up, and among them, 44 patients died, resulting in a mortality rate of 48.35%. Kaplan–Meier survival analysis revealed that the mortality rate in the ACC group was significantly higher than that in the no-ACC group (χ2 = 35.993, *P* < 0.001) (Fig. 2). Univariate Cox regression analysis demonstrated that low Kt/V, ACC, advanced age, longer duration of dialysis, comorbid diabetes, comorbid hypertension, and high SOST levels were all associated with all-cause mortality in the patients. Multivariate Cox regression analysis showed that Dialysis Time (hazard ratio [HR] = 1.012, 95% Confidence Interval [CI]: 1.029–3.708, *P* = 0.040), comorbid diabetes (HR = 1.954, 95% CI: 1.029–3.708, *P* = 0.040), and AAC (HR = 3.666, 95% CI: 1.333–10.081, *P* = 0.012) were identified as risk factors for all-cause mortality in peritoneal dialysis patients (Table 3).

**Table 3:** Cox regression analysis of overall mortality in patients with Parkinson’s disease

Items	Univariate analysis		Multivariate analysis	
	HR(95%CI)	*P*	HR(95%CI)	*P*
Female	1.673(0.887–3.157)	0.112		
Age	1.059(1.026–1.092)	< 0.001*		
Dialysis time	1.020(1.011–1.030)	< 0.001*	1.012(1.001–1.023)	0.041*
Calcium	0.764(0.166–3.516)	0.730		
Phosphorus	1.561(0.842–2.894)	0.157		
Albumin	1.022(0.916–1.142)	0.693		
Triglycerides	0.957(0.698–1.313)	0.786		
Cholesterol	0.817(0.580–1.150)	0.246		
ALP	1.002(0.993–1.011)	0.634		
LDL	0.729(0.446–1.190)	0.206		
Hemoglobin	1.000(0.999–1.001)	0.566		
iPTH	1.001(0.999–1.002)	0.399		
Kt/V	0.256(0.074–0.879)	0.030*		
Diabetes	3.343(1.812–6.169)	< 0.001*	1.954(1.029–3.708)	0.040*
Hypertension	2.746(1.481–5.091)	0.001*		
SOST	1.002(1.000–1.004)	0.014*		
BMI	0.970(0.862–1.094)	0.626		
AAC	7.599(3.506–16.470)	< 0.001*	3.666(1.333–10.081)	0.012*

*ALP* Alkaline phosphatase, *LDL* Low-density lipoprotein, *iPTH* intact parathyroid hormone, *Kt/V* urea clearance index, *SOST* sclerostin, *BMI* body mass index, *AAC* abdominal aortic calcification, *HR* Hazard Ratio, *CI* Confidence Interval

^*^with significant differences

## Discussion

The occurrence of vascular calcification (VC) is high in dialysis patients, with reported rates of 65% in peritoneal dialysis (PD) and 80–85% in hemodialysis (HD) patients, showing varying degrees of coronary or aortic calcification [11–13]. This is associated with the total duration of dialysis, with HD patients experiencing a 15% increased risk of VC per year. Even in young HD and PD patients aged 20–30, the prevalence of coronary artery calcification (CAC) remains over 80%. In this study, the incidence of abdominal aortic calcification (AAC) in our PD patients was found to be 50.55%.

Abnormal bone metabolism and vascular calcification are common and important contributors to increased mortality rates in patients [14]. As CKD progresses, significant changes occur in the bone and mineral hormone axis, leading to alterations in bone turnover and clinical manifestations such as decreased bone mass, increased bone fragility and fractures, as well as the occurrence of related vascular and valvular calcification, all of which have a significant impact on cardiovascular and long-term prognosis outcomes [12, 13]. Vascular calcification is considered to be an actively regulated process similar to cartilage and bone formation, although the signaling pathways involved are not fully understood [11]. Vascular calcification and cardiovascular disease are associated with chronic kidney disease-mineral and bone disorder (CKD-MBD), which is a key pathogenic factor leading to vascular calcification in cardiovascular disease. Previous studies have shown a potential association between serum SOST levels and the occurrence of vascular and soft tissue calcification in CKD patients. Vascular calcification is also believed to be a progressive osteogenic process triggered by arterial inflammatory substances. Therefore, these findings have led some to speculate that SOST may also play a role in the process of vascular calcification [15]. In this study, logistic regression analysis was used to analyze the factors influencing the occurrence of AAC in PD patients, and the results also suggested a significant role of SOST (OR = 1.006, 95% CI: 1.000–1.011, *P* = 0.038) in the development of AAC in PD patients.

Sclerostin (SOST) is a protein produced by bone cells and plays an important regulatory role in bone metabolism [16]. Under normal conditions, Sclerostin regulates bone metabolism by inhibiting the process of bone formation. As an inhibitor of the Wnt signaling pathway, it suppresses the differentiation of osteoprogenitor cells into osteoblasts, thereby inhibiting the formation of new bone [17]. However, in chronic kidney disease, the expression levels of Sclerostin are often elevated, particularly during or after CKD stage III [18]. This phenomenon may be related to multiple factors, including disturbances in phosphate metabolism due to impaired renal function, renal tubular injury, and abnormalities in parathyroid gland function [19, 20]. This study also suggests that SOST is a risk factor for vascular calcification in PD patients (OR = 1.006, 95% CI: 1.000–1.011, *P* = 0.038). Although the role of Sclerostin in chronic kidney disease is not fully understood, previous research has indicated that inhibiting Sclerostin may be a potential strategy for treating skeletal and vascular complications associated with chronic kidney disease.

In a previous study involving non-CKD patients, it was found that serum Sclerostin levels were significantly elevated in patients with atherosclerosis, vascular calcification, and valvular calcification [21]. In an animal model, ZhuD confirmed the association between Wnt signaling and arterial and valvular calcification through tissue analysis of calcified vessels in mice. The expression of Sclerostin increased in vascular smooth muscle cells during calcification, and Sclerostin may exert its biological function of inhibiting bone formation and vascular calcification by reducing osteoblast activity [22]. Sclerostin has been found in atherosclerotic plaques and may be involved in the development of atherosclerosis and vascular calcification [23]. Morales-Santana et al. [24] also reported Sclerostin as an independent correlate of intima-media thickness in carotid arteries. Furthermore, a study found the expression of Sclerostin in the calcified arteriovenous fistula in maintenance hemodialysis patients [25]. These findings indicate that Sclerostin plays a crucial role in the process of vascular calcification.

A study involving Chinese patients showed a positive correlation between levels of Sclerostin and carotid intima-media thickness (CIMT) in a cohort of 84 hemodialysis patients [26]. Another study selected 50 Parkinson’s disease patients undergoing peritoneal dialysis (PD) to investigate the relationship between Sclerostin levels and arterial stiffness. The results showed a significant correlation between the severity of arterial stiffness and high levels of Sclerostin in PD patients, indicating that elevated serum Sclerostin levels are a risk factor for the progression of atherosclerosis [27]. In a study by Qureshi et al. [28], 89 ESRD patients who underwent kidney transplantation were evaluated for the extent of vascular calcification (VC) using abdominal aortic and coronary artery calcification (CAC) scores. Serum Sclerostin levels were measured, and the results showed a significant correlation between high Sclerostin levels and the severity of VC (*P* = 0.003). In another study by Wang et al. [29], 161 CKD patients were included, and abdominal aortic calcification (AAC) scores assessed by abdominal X-ray were used to classify the patients. The results showed a positive correlation between higher Sclerostin levels and AAC (*P* < 0.001). Morena et al. [30] investigated the relationship between Sclerostin levels and CAC scores in 241 pre-dialysis CKD patients. After adjusting for confounding factors, higher Sclerostin levels were significantly associated with the presence of CAC (OR = 2.18, 95% CI: 1.06–4.49, *P* = 0.03).

Previous studies have focused on the prevalence and progression of aortic arch or coronary artery calcification and investigated their relationship with mortality rates [31–33]. In a longitudinal study spanning 10 years, hemodialysis patients with rapid progression of vascular calcification had a higher risk of death over time [34]. Multivariable Cox regression analysis in this study showed that AAC, longer dialysis vintage, and the presence of diabetes were independent risk factors for all-cause mortality in dialysis patients, consistent with previous research. In a recent study by YunZou et al. [35], low Sclerostin levels were found to be associated with better overall survival in PD patients. A meta-analysis by Kanbay et al. [36] included 9 observational prospective studies involving 1,788 patients. The meta-analysis concluded that there was no significant association between Sclerostin and all-cause mortality or cardiovascular mortality. In our study, after adjusting for dialysis vintage, vascular calcification, and comorbidities, no association was found between Sclerostin and survival in PD patients. Our research findings have important implications for clinical practice. Identifying these risk factors helps identify high-risk PD patients and implement appropriate interventions to prevent or delay the occurrence of vascular calcification. Strategies targeting the regulation of Sclerostin levels may help mitigate vascular calcification and improve patient outcomes. Further research is needed to elucidate the specific mechanisms by which Sclerostin influences vascular calcification and to evaluate the efficacy of interventions targeting Sclerostin in clinical practice.

### Limitation

This study has several limitations. Firstly, it employed a retrospective design, relying on existing medical records and imaging data. This design may lead to limitations in information retrieval and inconsistency in data quality. It also cannot control the allocation of interventions and may have potential confounding factors. Secondly, although the study followed up the enrolled patients for up to 48 months and recorded all-cause mortality, detailed follow-up data were lacking, preventing understanding of specific causes of death and other clinical outcomes. Thirdly, limited sample size with risk of overfitting when including too many variables in multivariable regression analysis. Fourthly, the absence of data on residual renal function. Additionally, the selection of study samples was limited to a specific time period and specific medical institutions, which may introduce selection bias and restrict the generalizability and external validity of the study results. In terms of measurement, the use of lateral abdominal X-ray as a common method to assess abdominal aortic calcification may be subject to subjective judgment and measurement errors by operators. Furthermore, retrospective studies are challenging to fully control for potential confounding factors such as genetic factors, nutritional status, and medication use. Lastly, although survival analysis and Cox regression analysis were conducted, causality between abdominal aortic calcification and all-cause mortality cannot be determined, and specific mechanisms were not deeply investigated. Therefore, future research should employ prospective designs, incorporate more extensive follow-up data and mechanistic studies to address these limitations and further advance research in this field.

## Conclusion

Longer dialysis duration, comorbid diabetes, comorbid hypertension, and SOST are risk factors for vascular calcification in PD patients. This study suggests that Sclerostin is an independent risk factor for vascular calcification in PD patients.

## Data Availability

Data is provided within the manuscript file.
